# Dissociable Components of Cognitive Control: An Event-Related Potential (ERP) Study of Response Inhibition and Interference Suppression

**DOI:** 10.1371/journal.pone.0034482

**Published:** 2012-03-28

**Authors:** Christopher R. Brydges, Karen Clunies-Ross, Madeleine Clohessy, Zhao Li Lo, An Nguyen, Claire Rousset, Patrick Whitelaw, Yit Jing Yeap, Allison M. Fox

**Affiliations:** 1 School of Psychology, University of Western Australia, Perth, Australia; 2 Neurocognitive Development Unit, University of Western Australia, Perth, Australia; Cuban Neuroscience Center, Cuba

## Abstract

**Background:**

Cognitive control refers to the ability to selectively attend and respond to task-relevant events while resisting interference from distracting stimuli or prepotent automatic responses. The current study aimed to determine whether interference suppression and response inhibition are separable component processes of cognitive control.

**Methodology/Principal Findings:**

Fourteen young adults completed a hybrid Go/Nogo flanker task and continuous EEG data were recorded concurrently. The incongruous flanker condition (that required interference suppression) elicited a more centrally distributed topography with a later N2 peak than the Nogo condition (that required response inhibition).

**Conclusions/Significance:**

These results provide evidence for the dissociability of interference suppression and response inhibition, indicating that taxonomy of inhibition is warranted with the integration of research evidence from neuroscience.

## Introduction

Cognitive control refers to the ability to selectively attend and respond to task-relevant events while resisting interference from distracting stimuli or prepotent automatic responses [1], [2]. Interest in the study of inhibitory processes has increased in the past two decades [3], reflecting the importance of inhibition in everyday cognition, and, “ultimately, for successful living” [4]. More recently, associations between inhibition and other executive functions, particularly updating and shifting have been studied in more depth [5], [6]. Despite a growing amount of research interest in the area [7], [8], there is still considerable debate as to the separability of the subprocesses of inhibition. Several theorists have proposed that, from a behavioural perspective, inhibition should be viewed as a group of separable, yet related, subprocesses [9], [10], [11].

Nigg proposed that there are four types of inhibition in cognitive psychology [11]; however, the present study focuses on only two of these: response inhibition, which involves the suppression of prepotent behavioural responses (as is required in a Go/Nogo task), and interference suppression, which is the active prevention of interference due to stimulus competition (such as that observed in a flanker task). Van Boxtel, van der Molen, Jennings, and Brunia [12] proposed an alternate, but not necessarily conflicting theory of inhibitory processing, where inhibition is classified as selective (i.e. an event in which a response has to be made, but is not prepotent) or nonselective, when no response is required [12]. This theory may be considered parallel to Nigg's taxonomy, as many tasks thought to measure response inhibition (such as Go/Nogo and stop-signal tasks) require nonselective inhibition, whereas tasks requiring interference suppression (such as Stroop and flanker tasks) require selective inhibition. However, a key difference between these processes is the time required for each process to be completed, as it is reasoned that selective inhibition takes longer due to it requiring discrimination; that is, on a forced-choice task, a choice still has to be made [13]. Although other prominent theories of inhibition [9], [10] use different terminology, they each converge upon the theory that inhibition refers to several related yet distinct processes, as opposed to a unitary construct.

Evidence from a variety of perspectives has been put forward in support of a unitary view of inhibition. From a behavioural perspective, Friedman and Miyake created latent variables of prepotent response inhibition and resistance to distracter interference and reported that that model fit was not significantly worse when the two variables were collapsed into one [8]. Verbruggen, Liefooghe, and Vandierendock used a combined flanker/stop-signal task to determine whether there was overlap between the processes of response inhibition and interference suppression [14]. They found that the stop-signal reaction time was longer for incongruous flanker trials than for congruous trials, suggesting a functional dependence between response inhibition and interference suppression. These results, together with the results from the confirmatory factor analysis [8], raise the possibility that a common inhibitory mechanism could be involved in both types of tasks [15].

Neuroimaging studies have associated successful inhibition with activation of regions of the prefrontal cortex and the right inferior frontal gyrus (IFG) [16], [17], [18]. Many studies using the event-related potential (ERP) have regarded the processes of response inhibition and interference suppression as similar constructs, reporting an enhanced anteriorly distributed negativity following presentation of stimuli that require inhibition of responses [19], [20], [21] or the suppression of conflicting information in flanker tasks [22], [23], [24], [25], although the amplitude and latency of the component varies across tasks [26], [27]. Bunge, Dudukovic, Thomason, Vaidya and Gabrieli examined the neural basis of response inhibition and interference suppression in adults and children with a hybrid Go/Nogo flanker task [28]. By incorporating the Go/Nogo stimuli into the flanker task, they reduced the potential effects of motivational differences and other non-specific task differences. The task was completed whilst functional magnetic resonance imaging (fMRI) was conducted, to determine unique patterns of neural activation associated with these two fundamental processes of cognitive control. The efficiency of interference suppression in adults (measured as the reaction time cost for incongruous trials relative to neutral trials) was associated with increased activation of the IFG/insula and the anterior portion of the middle frontal gyrus in the right hemisphere. Despite robust activation of the prefrontal, cingulate, and parietal cortices associated with the Nogo trials, none of these regions were significantly correlated with the efficiency of response inhibition (measured as the accuracy cost for Nogo trials relative to neutral trials). They attributed this result to the lack of variability in both behavioural performance and activation levels across individuals. One potential difficulty with the hybrid task developed by Bunge et al. is that the flankers provided the cue to inhibit responses and therefore required participants to consciously attend to these elements of the stimuli, rather than strategically suppress processing of these elements [29], [30]. This factor may have changed the way participants processed the incongruous stimuli, a conclusion that is supported by the low error rates observed following presentation of incongruous stimuli. Another potential difficulty is the lack of variability in behavioural and neural indices of response inhibition. Falkenstein, Hoormann and Hohnsbein reported that participants who performed worse on a Go/Nogo task had significantly smaller N2 amplitudes than those who performed well [31], suggesting that this neural index of inhibitory processing shows sufficient between-subject variability and could provide a more sensitive measure of online inhibitory processing.

The present study examined the dissociability of the two fundamental components of cognitive control, namely response inhibition and interference suppression, by recording the brain's electrical response to stimuli presented in a hybrid Nogo/flanker task. Two hypotheses were made: first, given that previous fMRI studies have reported differences in the regions of neural activation associated with response inhibition and interference suppression [28], [32], it was hypothesised that the amplitude of the N2 elicited in response to incongruous stimuli would be maximal at different scalp sites to that of the Nogo stimuli. Second, it was hypothesised that the latency of the N2 elicited in response to incongruous stimuli would be significantly longer than the latency of the N2 elicited in response to Nogo stimuli.

## Methods

### Ethics Statement

Approval for the study was provided by the Human Research Ethics Office of The University of Western Australia, and all participants provided written informed consent.

### Participants

Fourteen third-year psychology students studying at the University of Western Australia completed the experiment, and data from two participants were excluded as their performance on the incongruous flanker stimuli did not exceed chance. The mean age of the final sample was 21.3 years (*SD* = 2.1; range = 19–26).

### Materials

**Figure 1:**
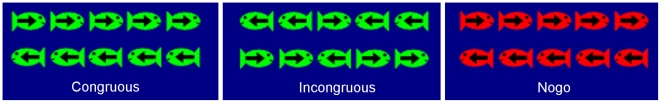
The six stimuli used in the present experiment.

A modified visual flanker task was used [33], [34]. Although this version is considered child-friendly, Rueda et al. found that this task produces results similar to the original flanker task [29] in adults [34]. Each stimulus consisted of five fish presented on a blue background. An arrow on the body of the fish indicated direction and the target was the central fish. Participants were instructed to press a response button on a keyboard (red felt patches on the ‘Z’ and ‘/’ keys) corresponding to the direction of the central fish. There were three conditions: in the congruent condition (.5 probability), the fish were green and all facing the same direction. In the incongruent condition (.25 probability), the fish were also green, however the flankers faced the opposite direction to the target. In the Nogo condition (.25 probability), the fish all faced the same direction but were all red, the participant was required to not respond. Each fish subtended .9° horizontally and .6° vertically, with .2° separating each fish (See Figure 1). Stimuli were presented in random order for 300 ms with a 2,000 ms inter-stimulus interval. The task was presented as a game in which the participants had to feed the hungry central fish. Speed and accuracy were equally emphasized. A practice block of eight trials was administered to ensure the participants understood the task requirements. A total of 176 trials were presented in one block.

### Electrophysiological Acquisition

The EEG was continuously recorded using an Easy-Cap™. Electrodes were placed at 33 sites (Fp1, Fp2, F3, F4, F7, F8, Fz, FC1, FC2, FC5, FC6, FCZ, FT9, FT10, C3, C4, Cz, T7, T8, CP1, CP2, CP5, CP6, P3, P4, P7, P8, Pz, PO9, PO10, O1, O2, Iz). Eye movements were measured with bipolar leads placed above and below the left eye. The EEG was amplified with a NuAmps 40-channel amplifier, and digitized at a sampling rate of 250 Hz. During recording, the ground lead was located at AFz and the right mastoid was set as reference, and an averaged reference was calculated offline. Prior to recording, impedances were below 5 kΩ. The ERP processing was conducted offline using Scan 4.3 software. Offline, the EEG recording was digitally filtered with a 1–30 Hz zero phase shift band-pass filter (12 dB down). The vertical ocular electrodes enabled offline blink reduction according to a standard algorithm.

### Data Analysis

Epochs encompassing an interval from 100 ms prior to the onset of the stimulus and extending to 1000 ms post-stimulus were extracted and baseline corrected around the pre-stimulus interval. Epochs containing artifacts larger than 150 µV or where an incorrect behavioral response was committed were excluded from the ERP average. The average number of trials included in each grand-averaged waveform was 84 trials for the congruous condition, 34 trials for the incongruous condition, and 41 trials for the Nogo condition. Participants committed between 0 and 12 false alarm responses following Nogo stimuli (*M* = 1.8, *SD* = 3.4). One missed trial following a congruous stimulus was recorded, and no misses were observed following incongruous stimuli. Between 0 and 5 incorrect responses were recorded following congruous stimuli (*M* = 1.8, *SD* = 1.9) and between 2 and 13 incorrect responses were recorded following incongruous stimuli (*M* = 8.6, *SD* = 3.6). Difference waveforms were then calculated by subtracting the individual ERP average elicited following presentation of the congruent stimuli from the ERP average elicited following presentation of the incongruent stimuli and the Nogo stimuli. We calculated the interval over which the N2 inhibition effect was significant by comparing the amplitude of the difference waveforms at each time point from 0–450 ms against a mean value of zero. To control for the number of comparisons conducted, we required a successive sequence of 11 statistically significant values based on an autocorrelation of 0.9 and graphical threshold of 0.05, as detailed by Guthrie and Buchwald [35]. In the incongruous difference waveform, the N2 effect was significant over the interval 356–408 ms at Cz. In the Nogo waveform, the N2 effect was observed considerably earlier, over the latency 256–300 ms, and the amplitude differed from zero at the Fz site only. As it was not possible to identify a clear peak in the waveform at each of the sites, the scalp topography of the N2 effects within each condition were examined by computing the mean amplitudes over the 356–408 ms latency interval for the incongruous - congruous difference waveforms and over the 256–300 ms latency interval for the Nogo - congruous difference waveforms. ANOVA with scalp site (Fz, FCz, Cz) as a repeated measures factor was conducted on the mean amplitudes extracted. Latency and amplitude of the N2 effect were quantified for the peaks within a 248–408 ms latency window at the site of maximal amplitude only. This window was chosen to capture the intervals identified in difference waveform analyses for both conditions and to ensure the maximum point was identified in each participant's waveform.

## Results

### Behavioral Results

Relative to the congruous condition, performance was impaired in the incongruous condition in terms of both response time (congruous stimuli *M* = 386 ms, *SD* = 20.9; incongruous stimuli *M* = 460 ms *SD* = 32.3; *F*(1,11) = 131.9, *p*<.001, η_p_^2^ = .92), and accuracy (congruous stimuli *M* = 97.9%, *SD* = 2.3; incongruous *M* = 80.5%, *SD* = 8.2; Nogo *M* = 95.8%, *SD* = 7.8).

### ERP Results

**Figure 2:**
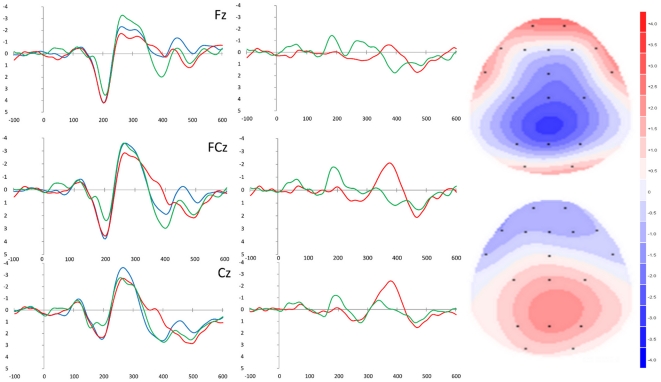
Stimulus-locked ERP waveforms, difference waveforms, and scalp topographic maps. Left-hand panel: Grand-averaged ERP in response to congruous (blue), incongruous (red), and Nogo (green) stimuli with the amplitude (µV) as the y-axis and time (ms) as the x-axis. Time 0 represents stimulus onset. Middle panel: Grand-averaged difference waveforms computed as the incongruous – congruous waveform (red) and Nogo – congruous (green). Right-hand panel: Topographic distribution of amplitude at the peak latency of the N2 identified in the difference waveforms (incongruous – congruous is shown in the upper map, and Nogo – congruous is shown in the lower map).

Figure 2 shows the stimulus-locked grand averaged waveforms for each condition and the difference waveforms computed by subtracting the ERPs elicited to congruous stimuli from each of the other two waveforms.

The amplitudes and latencies of the N2 peak identified in the difference waveforms are summarized in Table 1.

**Table 1:** N2 Amplitude and Latency Summary Statistics (Means, with Standard Deviations in Parentheses).

Condition	Site	N2 mean amplitude	N2 peak amplitude	N2 peak latency
Incongruous – Congruous	Fz	−0.4 (1.6)	-	-
	FCz	−1.6 (1.9)	-	-
	Cz	−2.0 (1.7)	−3.3 (1.9)	379 (23)
Nogo - Congruous	Fz	−0.9 (0.9)	−1.9 (1.1)	288 (33)
	FCz	0.1 (1.6)	-	-
	Cz	0.8 (1.7)	-	-

The negativity observed in the incongruous – congruous difference waveform over the 356–408 ms interval was centrally distributed (quadratic trend, *F*(1,11) = 6.5, *p* = .027; Cz>FCz>Fz). In contrast, the negativity observed in the Nogo – congruous difference waveform over the 256–300 ms interval was frontally distributed (linear trend, *F*(1,11) = 17.0, *p* = .002, Fz>FCz>Cz).

The negativity observed in the incongruous – congruous difference waveform peaked significantly later than the negativity observed in the Nogo – congruous difference waveform (*F*(1, 11) = 75.2, *p*<.001, *d* = 2.6).

## Discussion

The results of this study showed that the topography and latency of the N2 were different following presentation of incongruous and Nogo stimuli. The N2 was found to be more frontal in the Nogo condition and more central in the incongruous condition, indicating that different neural generators are responsible for the two processes. Topographical differences between the response inhibition ERP effect and the interference suppression ERP effect suggest that these two processes originate in different neural regions, although source analysis would be required to verify this conclusion. While topographical differences do support a suggestion that non-equivalent cognitive processes are engaged, it is very possible that there is a common set of generators that differentially contribute to each process. Previous neuroimaging studies have suggested the dorsolateral prefrontal cortex is involved in response inhibition [36], [37], whereas the anterior cingulate cortex, located in a more central region, is implicated in the suppression of interference [38], [39]. These differences add to the experimental evidence examining whether these two aspects of inhibition reflect a unitary construct, or separable processes.

Latency was significantly longer in the incongruous condition than the Nogo condition, indicating that successful interference suppression required more time than successful response inhibition in our task. Although Friedman and Miyake speculated that interference suppression occurred before response inhibition [8], the current study found the opposite to be the case. Specifically, Friedman and Miyake reasoned that interference suppression seems to refer to a stage of perceptual processing that occurs very quickly, and response inhibition is synonymous with a later stage of processing in which a motor response must be modified or withheld. However, results from the current study suggest that response inhibition occurs before interference suppression. One possible explanation for this is the differences between ‘stop’ and ‘change’ paradigms within inhibition [12], [13]. That is, response inhibition requires (in the case of the Nogo condition) the participant to inhibit all responses, whereas interference suppression requires participants to make a response despite interference from distractor stimuli. Conceptually, response inhibition can be viewed as a complete ‘shut-down’ of all responses, whereas interference suppression involves a more specific shut-down of processes, resulting in a more complex and intricate procedure, requiring more time to occur successfully. An alternate explanation for the differences in latency may be the use of color as the distinguishing feature of the Nogo stimuli. Previous research suggests that color processing occurs prior to the processing of form [40]. Given that in the current study the cue to inhibit the response was based on the color of the stimuli whereas the interference suppression was cued by form, the initiation of response inhibition would occur before the interference suppression mechanism.

There is still some debate about the underlying mechanisms of the flanker task. An opposing explanation to the cognitive control account of the flanker task is the grouping effect [41], whereby feature similarity of several objects in an array is thought to cause all the objects to be grouped, such as in the congruous condition. This grouping effect may account for enhanced behavioral performance in the congruous condition compared to the incongruous condition, in which the target stimulus faces the opposite direction to the flankers, disrupting the grouping mechanism. However, the presence of the N2 difference waveform suggests that there is some degree of interference suppression occurring in the incongruous condition [31], supporting the role of cognitive control within the flanker task.

The results of this study could contribute to the development of new interventions in psychopathology. Previous research in children has found that Attention Deficit Hyperactivity Disorder is related to diminished response inhibition [42], whereas interference suppression is reportedly unimpaired [43]. The opposite profile of inhibitory deficits has been reported in children with autism spectrum disorder. Children with autism spectrum disorder were found to have impaired interference suppression, but intact response inhibition [44]. In light of the current electrophysiological results and prior fMRI research indicating distinct anatomical substrates for these two processes, future research using these techniques could contribute to our understanding of the underlying neural abnormalities in these clinical conditions.

The present study examined the dissociability of two domains identified within the taxonomy of inhibitory processes proposed by Nigg [11] using ERP techniques which have high temporal resolution, but with lower spatial resolution than other cognitive neuroscience techniques such as fMRI. Future research would be needed to tease apart the differences between Nigg's taxonomy [11] and van Boxtel et al.'s theory of selective and nonselective inhibition [12].

In conclusion, research integrating modern cognitive neuroscience techniques in addition to behavioral measures has highlighted the ways in which two aspects of inhibitory processes previously regarded as representing a unitary construct [8] can be dissociated. Further development of taxonomy of inhibition [9], [10], [11] would benefit from the continued integration of research evidence derived from these neuroscientific methods.
